# Positron Emission Tomography—Computer Tomography Scan Used as a Monitoring Tool Following Cellular Therapy in Cerebral Palsy and Mental Retardation—A Case Report

**DOI:** 10.1155/2013/141983

**Published:** 2013-02-03

**Authors:** Alok Sharma, Hemangi Sane, Amruta Paranjape, Nandini Gokulchandran, Pooja Kulkarni, Anjana Nagrajan, Prerna Badhe

**Affiliations:** ^1^Department of Medical Services and Clinical Research, NeuroGen, Brain and Spine institute Private limited, Surana Sethia—Hospital and Research Centre, Suman Nagar, SionTrombay Road, Chembur, Mumbai 400071, Maharashtra, India; ^2^Department of Research and Development, NeuroGen, Brain and Spine institute Private limited, Surana Sethia—Hospital and Research Centre, Suman Nagar, SionTrombay Road, Chembur, Mumbai 400071, Maharashtra, India; ^3^Department of Neuro-Rehabilitation, NeuroGen, Brain and Spine institute Private limited, Surana Sethia—Hospital and Research Centre, Suman Nagar, SionTrombay Road, Chembur, Mumbai 400071, Maharashtra, India; ^4^Consultant Neuropathologist, Department of Medical Services and Clinical Research, NeuroGen, Brain and Spine institute Private limited Surana Sethia—Hospital and Research Centre Suman Nagar, SionTrombay Road, Chembur Mumbai 400071, Maharashtra, India

## Abstract

Cerebral palsy (CP) is one of the non-progressive neurological diseases caused by damage to the brain tissue at birth, which leads to physical, cognitive and perceptive symptoms. Even after lifelong medical and therapeutic management there are residual deficits which affect the quality of life of the patients and their families. We examined a maximally rehabilitated, 20 year old male suffering from CP and Mental Retardation (MR). He had diplegic gait and Intelligence Quotient (IQ) score of 44 with affected fine motor activities, balance, speech and higher functions. Positron Emission Tomography—Computer Tomography (PET-CT) scan identified frontal, temporal, parietal, occipital, left cerebellar lobes, amygdala, hippocampus, and parahippocampus as the affected areas. He was treated with cellular therapy of Autologous Bone Marrow Derived Mono-Nuclear Cells (MNCs) transplantation followed by multidisciplinary rehabilitation. Six months following therapy, PET-CT scan showed significant increase in metabolic activity in all four lobes, mesial temporal structures and left cerebellar hemisphere, also supported by clinical improvement in IQ, social behavior, speech, balance and daily functioning. These findings provide preliminary evidence to support the efficacy of cellular therapy for the treatment of CP with MR. PET-CT scan can also be viewed as an impressive tool to monitor the effects of cellular therapy.

## 1. Introduction 

Cerebral palsy (CP) is defined as, “*a group of disorders of the development of movement and posture, causing activity limitation, that are attributed to nonprogressive disturbances that occurred in the developing fetal or infant brain. The motor disorders of cerebral palsy are often accompanied by disturbances of sensation, cognition, communication, perception, and/or behaviour, and/or by a seizure disorder.*” It has been further classified into various groups based on the motor abnormalities, associated impairments, anatomical and radiological changes, causation, and timing [1]. Mental retardation (MR) or intellectual developmental disorder is defined as per the International Classification of Diseases (ICD) working group as, “*a group of developmental conditions characterized by significant impairment of cognitive functions, which are associated with limitations of learning, adaptive behavior and skills* [2].” MR is usually classified on the basis of IQ scores [3]. MR was observed to coexist in 30% of the cases of CP in full term infants [4]. MR and CP have some common etiological factors [5, 6]. Both limit function and affect Quality of Life (QoL) [7]. The current management of CP constitutes of pharmacological management [8, 9] Surgical management [10–12] physiotherapy, occupational therapy, speech therapy, and other interventions [7] targeted to manage these symptoms. Due to inability of the neurons to instinctively repair themselves [13], there is no cure for cerebral palsy as yet. Stem cells however are capable of trans-differentiation into various mature tissue types [14]. Stem cells can be obtained from various body tissues [15]. For the purpose of this case we have used adult autologous bone-marrow-derived Mononuclear cells (MNCs). 

Positron emission tomography (PET)-computer tomography (CT) scan as an outcome measure to assess the effect of stem cells on CP has rarely been used [16, 17]. This case study therefore highlights the PET-CT scan changes and explores the clinical effects of transplantation of bone marrow MNCs in our patient with CP and MR. 

## 2. Case Report

A 20-year-old male with CP and MR was admitted after reaching a plateau with other treatments. Patient was born full term by C-section. He suffered a hypoxic injury at birth due to umbilical cord wrapping around the neck. Normal milestones were achieved on time till eight months, when he suffered a febrile convulsion, after which he had delayed milestones. The last episode of convulsions was at the age of eleven months. He was diagnosed with cerebral palsy with mental retardation. He started with regular physiotherapy treatment since the age of 1 year and has been attending special school till now. He started walking at the age of 5 years and there was some development of speech by the age of 7 years.

### 2.1. Assessment

At the time of admission he was hypertonic (Modified Ashworth Scale—Grade I for Bilateral Lower Extremities) and hyperreflexic (all the tendon reflexes were brisk). He had voluntary control of his lower extremities and walked using a cane in a diplegic gait. He had sensation and voluntary control of bladder and bowel. In standing his knees showed flexion attitude whereas feet showed valgus attitude. Hand movements were voluntary but clumsy. He needed minimal assistance for most of his ADL with Functional Independence Measure (FIM) score as 89. He was oriented to time, place, and person. Concentration, eye contact, and social skills were poor. He had slurred speech, irrelevant talking, and no dysphagia. He mainly communicated verbally with simple sentences. He understood simple unidirectional commands. He could only answer questions related to recent memory and memory related to names was poor. Mental status examination and Intelligence Quotient (IQ) test suggested moderate mental retardation with an intelligence quotient with an IQ score of 44. 

PET-CT scan showed no structural abnormality and diffuse functional abnormality as evidenced by reduced fluorodeoxyglucose (FDG) uptake in frontal, temporal, parietal, occipital lobes and left cerebellar lobes. Temporal Mesial structures showed pronounced reduction in FDG uptake in the regions of amygdala, hippocampus, and parahippocampal gyri.

### 2.2. Outcomes Used

FIM was used to determine the functional independence, IQ score to ascertain the level of MR and positron emission tomography (PET)-computer tomography (CT) of brain to assess the functional and structural changes.

### 2.3. Material and Methods

Patient selection was based on the World Medical Associations Helsinki declaration [18]. An evidence-informed protocol was designed. This protocol was reviewed and approved by Institutional Committee for Stem Cell Research and Therapy (IC-SCRT) in accordance with the Indian Council of Medical Research (ICMR) guidelines. After an informed consent we treated the patient with cellular therapy followed by rehabilitation. The aim was to make the patient self-dependent reducing the impairment and improving function. Patient underwent serological, biochemical and hematological blood tests, PET-CT scan of brain, Magnetic Resonance Imaging (MRI) plus diffusion tensor imaging of brain, chest X-ray, electroencephalography, and electrocardiography a week before adult autologous bone marrow MNCs transplantation. 

Granulocyte colony stimulating factor (GCSF) was administered 48 hrs and 24 hrs before the transplantation of bone marrow MNCs. On the day of transplantation as the patient lay in supine position, local anesthesia was administered in the region of the right anterior superior iliac spine. 100 mL of bone marrow was aspirated using the bone marrow aspiration needle and collected in heparinized tubes. The aspirate was then transferred to the laboratory. In the stem cell laboratory the MNCs were separated by the density gradient method. The cells were sent for CD34^+^ counts by Fluorescence activated cell sorter (FACS) analysis with the viability of 98%. The cells were injected intrathecally through an epidural catheter at the level of L4-L5. Number of cells injected were, 10^6^ times the exact body weight of the patient. Simultaneously Methyl Prednisolone 1 gm in 500 mL Ringer Lactate was given intravenously.

Neurorehabilitation therapy was a multidisciplinary rehabilitation protocol including physiotherapy, occupational therapy, speech therapy, psychotherapy, and diet advice. Physiotherapy treatment aimed at progressive resistive strength training for the weak muscles [19], stretching exercises for the tight muscles, balance training, and functional training [20]. Occupational therapy aimed at functional training for carrying out activities of daily living (ADL) and behavior training to improve social participation [21]. Speech therapy was given to improve speech complexity and increase comprehension [22]. Cognitive therapy was also included. He was treated with art therapy and play therapy and alphabet and number worksheets were given to complete. Psychological counseling was conducted for the family members.

We advised to continue supervised therapy at home. He was followed up at 3 months and 6 months post intervention. At 6 months post intervention a detailed reassessment was carried out and repeat PET-CT scan was obtained. At 12 months post intervention patient was evaluated to assess the prognosis. 

### 2.4. Results

At 3 months followup improvement was noted in attention, concentration, and eye contact. He was following complex commands. The quality of fine motor activities had improved. He could hold and eat food without dropping. At 6 months followup clarity of speech had improved significantly. He was now able to perform one leg standing due to improved balance. He was minimally dependant for the ADL. FIM score improved from 89 to 93. Socially he had started interacting with others and was cooperating with parents, at social gatherings. The IQ score improved from 44 to 55. His appetite had improved and he had gained 6 kgs in the last 6 months. At 12 months followup there was further improvement in attention, concentration, and eye contact. He was able to maintain the eye contact for the entire length of the conversation. Following commands had improved. He would now pick up and bring the required object from various scattered objects. His IQ score was maintained at 53. Motor coordination, gross and fine motor control had improved as which could be noted in various day to day activities. He could walk independently without support and could perform tandem staircase climbing with minimal support. He could participate in leisure sports with his peers. At school, teachers reported a significant increase in Positive Peer Interaction. 

**Figure 1:**
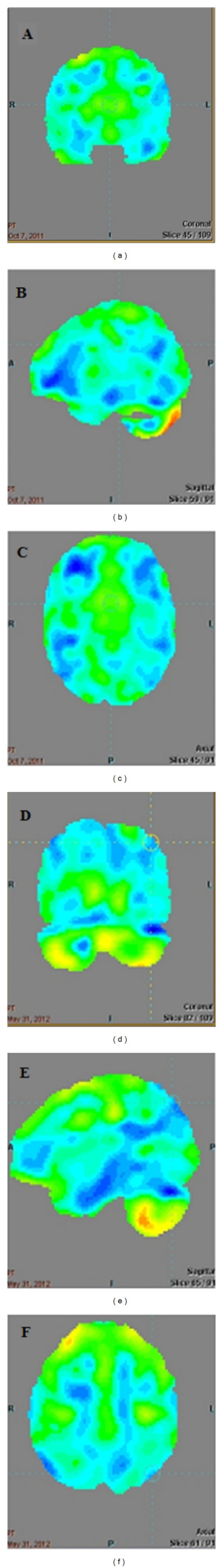
PET-CT scan images prestem cell therapy ((a), (b), (c)) and poststem cell therapy ((d), (e), (f)) showing the increased FDG uptake in the frontal, parietal, temporal, occipital, mesial temporal structures bilaterally and left cerebellum.

**Figure 2:**
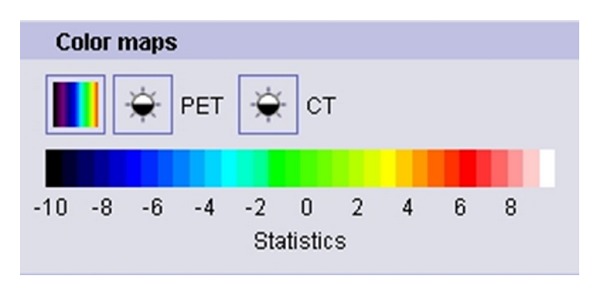
French Color coding for statistical reconstruction of the PET-CT scan images.

The PET-CT scan when compared with the previous scan showed that the uptake of FDG had increased significantly in frontal, temporal, parietal, and occipital lobes. Increased FDG uptake was seen in bilateral basal ganglia as well as the mesial temporal structures like bilateral middle and posterior cingulategyri, the left amygdala, right parahippocampalgyrus, and the left cerebellar hemisphere (Figures 1 and 2).

## 3. Discussion 

CP is a nonprogressive neurological disorder. The prognosis of CP depends on the sensory, motor, perceptive, and cognitive impairment caused by the extent of brain damage. These symptoms, in absence of any concomitant neurological disorder, when aggravated are not directly associated with any further damage to the central nervous system [23]. The central nervous system is unable to repair the damaged tissue with the help of existing treatment options [13]. The mainstay of the management of cerebral palsy is to maintain the function and reduce the impairment, utilizing neural plasticity to develop new motor-learning pathways. The management also aims at making use of assistive devices and functional aids to make the patients functionally independent to be able to participate socially [7–14, 19–22].

In our case study we treated a patient suffering from CP and MR. The management was multidimensional to address various impairments. However along with managing the impairments we attempted to stimulate the nervous system to repair itself. This was achieved with the help of the bone marrow MNCs transplantation. Safety of bone marrow MNCs has been demonstrated in various earlier trials [24, 25]. Using autologous cells reduces the possibility for immune rejection [26] and is found to be more effective than allogenic cells [27]. Intrathecal application ensures focused application into the central nervous system. CSF also harbors properties which support cell growth [28]. The stem cells are believed to possess special properties of self renewal as well as clonogenic properties. This allows them to grow into different cell types from various germ layers [29–31]. Stem cells also possess the property of dedifferentiation, where in the differentiation potential is increased by conversion of the cells into a more primitive phenotype with altered gene expressions [32]. The stem cells also bring about changes in the surrounding tissue either altering the micro- or macroenvironments of the damaged tissue. These paracrine effects include changes in the internal repair process of axons and myelin [33], immunemodulation [34], secretion of various growth factors, secretion of vascular endothelial growth factor (VEGF), angiogenesis [35, 36], regulation of cell apoptotic process [37], reduction of inflammation [38], and activation of neighboring stem cells [39].

Patient in this case study had attained a plateau with other pharmacological, surgical, and rehabilitative therapies. We treated him with a multidisciplinary approach consisting of adult autologous bone marrow derived MNCs transplantation and rehabilitation in the hospital for 6 days, followed by a home program. Cell transplantation followed by rehabilitation and increased physical activities has been suggested to be more effective than either of the therapies alone [40]. In our case report following this protocol we observed favorable changes functionally and in the PET scan which showed increased fluorodeoxyglucose (FDG) uptake and retention in various areas of brain. The basic principle of functional neuroimaging is that the changes in the blood flow and the energy metabolism is associated with the activity of the nervous tissue [41]. The FDG, which is an analogue of glucose, is used to measure this metabolic activity of the tissue. Glucose transporter proteins transport FDG to the cells. It undergoes common metabolic changes as that of glucose molecules; however once it has been converted to FDG-6-phosphate it cannot be further metabolized. Because the cell membrane is impermeable to this molecule it gets trapped in the cell [42]. This trapping is directly proportional to the rate of glycolysis in the tissue. Glycolysis is a metabolic pathway used to release energy from glucose molecule. PET measures the retention of FDG per predetermined volume, standard uptake value (SUV). Standard uptake value is the ratio of the actual concentration of glucose in brain tissue and the hypothetical concentration of the glucose in brain tissue if it was distributed evenly in all the areas of brain. SUV is calculated for specific region of interest (ROI) based on the image acquisition and is only the best estimate of the absolute uptake [43]. Increased SUV indicates better metabolic activity of the tissue [44]. Use of SUV as an outcome measure has been debated by various authors, as it is significantly influenced by various technical, biological and physical factors and parameters [43, 45, 46]. PET-CT scan therefore is not recommended as a diagnostic tool. However, PET has previously been used for monitoring the progress with a particular treatment in cerebral palsy [47] and it has also been used to differentiate various types of cerebral palsy from one another [48]. We followed robust protocol based on the European Association of Nuclear Medicine (EANM) guidelines [49] for administration of radiotracer, PET measurement, and image reconstruction to reduce the effect of various confounding factors that affect brain metabolism, image reconstruction, and SUV. This ensured the pre- and posttherapy comparability of the PET-CT scans. As per the EANM guidelines the interpretation was based on the SUVs, visual interpretation of the absolute and statistical reconstruction of the image, and value of standard deviations away from the mean as compared to baseline data.

The patient showed increased motor control of various gross and fine motor activities and clarity of speech at the six months followup. Parents also reported more responsible social participation and co-operation in following commands at social gatherings. These changes can be associated with the improvements in the frontal lobe functioning as identified on the PET scan. Temporal lobe which is grossly responsible for understanding auditory cues and language showed increased FDG uptake which can be correlated to his ability of being able to follow more complex commands. Various parts of limbic system also showed increased FDG uptake which could be reflected as increased appetite and subsequent weight gain, more socially responsible behavior, and increased social participation. Increased FDG uptake in mesial temporal structures correlates with enhanced memory. Finally the balance improvements and improvements in the fine motor activities can be attributed to the increased function of cerebellum as reflected in the PET scan. The interpretation of the PET-CT scan changes also corelated with the clinical improvement in our patient. 

Functionally the patient showed an improvement of 4 points on the FIM scale. This change is not Minimal Clinically Important Difference (MCID) [50]. However it was also observed that lower the FIM score at the time of admission the higher is the change in FIM scale. As the patient we treated had a significantly independent functional status the FIM score did not show a greater change at the end of our followup. The significant change however was observed in the IQ score which increased from 44 to 55. This was noted clinically as a more responsible social participation and ability to follow more complex verbal commands and improved balance and muscle performance [51]. 

It is observed that cellular therapy can be safe and effective in repairing the damage to the nervous tissue. This case study further supports the observation with objective PET-CT scan findings and provides preliminary evidence suggestive of the efficacy of cellular therapy in treatment of CP and MR. However, it is important to gather more evidence making use of randomized controlled trials with a larger sample size. PET-CT scan can also be viewed as an impressive tool to monitor the effects of cellular therapy, although a vigorous protocol is necessary.
